# The Childbirth Experience Survey (CBEX): An Analysis of Qualitative Survey data

**DOI:** 10.1007/s10995-025-04043-4

**Published:** 2025-02-05

**Authors:** Samia Saeb, Lisa M. Korst, Ferina Farahnik, Jeanette McCulloch, Naomi Greene, Moshe Fridman, Kimberly D. Gregory

**Affiliations:** 1 Department of Obstetrics and Gynecology, Cedars-Sinai Medical Center, 8700 Beverly Blvd. West Tower, Los Angeles, CA USA; 2 Childbirth Research Associates, LLC, North Hollywood, CA USA; 3 Birthswell, Ithaca, NY USA; 4 AMF Consulting, Los Angeles, CA USA; 5 Departments of Obstetrics and Gynecology and Community Health Sciences, David Geffen, School of Medicine, University of California, Los Angeles, USA

**Keywords:** Childbirth, Patient-reported outcomes, Patient experience, Qualitative research, Quality of care

## Abstract

**Background:**

In collaboration with community research partners, a national cross-sectional online Childbirth Experience Survey (CBEX) of pregnant and postpartum birthing people was administered in 2016. The linked antepartum-postpartum survey included items across 18 domains (e.g., labor management, pain management, newborn care and feeding), and identified 23 childbirth-specific postpartum patient-reported outcomes (PROs) that were associated with hospital satisfaction. CBEX was implemented in 16 California hospitals to identify hospital-specific opportunities for improvement in care. We analyzed postpartum qualitative survey responses (1) to evaluate the content validity to test the representativeness of existing CBEX domains, (2) to assess for any potential new domains or topics of interest within existing domains, and (3) to use these data to provide hospitals with actionable information for practice improvement.

**Methods:**

This was an analysis of qualitative survey data based on the following CBEX item: “Is there anything else you would like to share about your birth experience?” Patients could provide multiple comments. Using Atlas.ti Version 8, we mapped participant responses to the 18 existing CBEX domains.

**Results:**

Of 525 surveys completed between Oct 2018 and Sept 2020, 172 patients responded to the qualitative item. A total of 235 comments were analyzed and all were able to be mapped to the 18 domains. Qualitative responses highlighted subtleties within several CBEX domains: (1) labor management: pressure from the care team to have a labor induction; (2) pain management: epidural effectiveness, timing, dosage, and education; (3) empathy and respect: issues related to students and residents; and (4) newborn feeding: rough physical handling of patients by nurses, specifically during lactation consults.

**Discussion:**

CBEX survey data is currently being utilized in hospitals to inform childbirth-specific quality improvement initiatives. By capturing detailed voluntary participant responses, CBEX provides the opportunity to document and explore nuanced aspects of the childbirth experience and subtleties that may be contributing to maternal dissatisfaction.

## Significance

What is Already Known on this Subject?

What this Study Adds?

Patient-reported outcome and experience (PRO) data have become a critical component of health services quality measurement. The Childbirth Experience Survey (CBEX) was developed to assist hospitals with identifying childbirth-specific quality improvement priorities and targets.

The study confirmed content validity of the existing 18 CBEX domains. CBEX qualitative data identified subtle and specific experiences and interactions within the domains of labor management, pain management, empathy and respect, and newborn feeding. By capturing detailed voluntary participant responses, CBEX provided the opportunity to document nuanced aspects of the childbirth experience and subtleties that may be contributing to maternal dissatisfaction.

## Introduction

Maternity care in the United States is a national priority (Hasbrouck, 2021; Office of the Surgeon, 2020). Maternal morbidity and mortality (MMM) are on the rise and disproportionately impacts birthing people of color, with Black, Indigenous, and other marginalized groups facing significantly higher rates of MMM compared to their White counterparts (Howell, 2018). These racial and ethnic disparities are known to be driven by systemic racism, structural inequalities, and social determinants of health (Crear-Perry et al., 2021; National Academy of Sciences, 2021).

To address the disparities in maternal health outcomes, a growing body of research advocates for comprehensive reforms in maternity care, including hospital-wide policies that promote equity, integrate midwives and doulas into care teams, and increase access to culturally sensitive care for all birthing people (National Academy of Sciences, 2021). Despite these calls to action, hospitals do not routinely capture or document the unique and complex experiences of birthing people, creating a critical gap in knowledge.

Documentation of the maternity care experience is becoming increasingly important as rates of maternal morbidity, dissatisfaction, and mistreatment before, during, and after pregnancy increase (Fink et al., 2023; Mohamoud et al., 2023). A more detailed and nuanced understanding of the patient experience in childbirth can assist hospitals and caregivers in implementing evidence-based interventions that align with the principles of respectful maternity care, which emphasizes treating birthing people with dignity, providing clear communication, and ensuring their participation in healthcare decisions (Vedam et al., 2019).

To capture the breadth and depth of the maternity care experience, the Childbirth Experience Survey (CBEX) was developed and validated in 2016 by the Childbirth Patient-Reported Outcomes Partnership (i.e., the Partnership) (Gregory et al., 2019). This is a group consisting of health services researchers (Maternal Quality Indicators Work Group), care providers, pregnant and postpartum people, advocates for pregnant people, hospital quality experts, and senior administrators. CBEX is a 2-part survey, and its administration is targeted to occur at both 36 gestational weeks and 8 weeks postpartum. The survey includes items across 18 childbirth domains, ranging from communication, empathy and respect, to pain assessment and newborn feeding. Part 1 specifically captures information related to a patient’s clinical history and values and preferences (V&P) and Part 2 captures patient-reported experiences and outcomes (PROs) (Korst et al., 2018; Gregory et al., 2019). CBEX also includes an unlimited text box at the end of the survey for participants to provide additional feedback about their birth experience.

The purpose of this study was to analyze the postpartum (Part 2) qualitative survey responses from the participants for the following purposes: (1) to evaluate the content validity to test the representativeness of existing CBEX domains, (2) to assess for any potential new domains or topics of interest within existing domains, and (3) to use these data to provide hospitals with actionable information for practice improvement.

## Methods

This study complied with all stipulations for human subjects research under Institutional Review Board (IRB) Pro00050845. All persons gave their informed consent prior to their inclusion in the study.

In 2018, CBEX was implemented in 16 California hospitals to identify variation in childbirth-specific PROs across hospitals (Saeb et al., 2023). For example, CBEX data identified variation and opportunity within the CBEX domains of communication (e.g., “No one explained to me what was going on.”) and pain management (e.g., “I experienced severe postpartum pain.”). Participating hospitals used these data to identify priorities for quality improvement (QI) initiatives and continue to collect CBEX data for tracking and trending QI initiatives.

### Participant Recruitment

Postpartum patients were recruited from 16 CBEX study hospital sites. Patients who were at least 8 weeks postpartum were eligible to participate. Recruitment strategies varied by hospital. Some patients learned about the survey in person during their clinic appointments, hospital-tour, or prenatal education classes. Others received virtual invitations via email and text message. The survey was hosted on a third-party platform that was compliant with HIPAA (Health Insurance Portability and Accountability Act). All patients were required to consent to participate and create a user account. Participants who completed the postpartum survey received a $5.00 electronic gift card.

### Response Coding

First, the individual qualitative survey responses were imported into ATLAS.ti Version 8 and a coding list of the 18 CBEX domains was created. Second, 2 authors; one primary author (SS) and a secondary author (FF) independently reviewed transcripts of qualitative responses from postpartum patients who responded to the question, “Is there anything else you would like to share about your birth experience?” Patients could provide multiple comments. The authors used a deductive dual coding approach, where each author independently mapped the individual responses to the 18 CBEX domains. Finally, the authors met to review each assigned code. Discordant codes were discussed, and consensus was reached between the 2 reviewers.

## Results

A total of 525 postpartum surveys were completed between October 30, 2018, and September 7, 2020. Of the completed surveys, 172 patients (33%) responded to the qualitative item (Table 1). Those with narrative vs. non-narrative responses did not differ with respect to race or Hispanic ethnicity, foreign birth, education, or completing the survey in English. Neither nulliparity nor the mode of delivery differed between the 2 groups. The only identified difference was in the proportion of respondents with private vs. public health insurance (79.1% vs. 62.0%, pairwise comparison *P* <.001). Notably, a substantial portion of respondents had consistently missing data across race, ethnicity, nativity, education and insurance.

**Table 1:** Summary of demographic characteristics among survey respondents with and without a narrative response (*N* = 525)

CBEX Variable	Narrative Response *N* = 172	No Narrative Response *N* = 353	Total Responses *N* = 525	*P*-Value
	N (%)	N (%)	N (%)	
**Race** White Black Asian Alaskan Native Native Hawaiian/PI More than one race Other Decline to answer Missing	36 (20.9%) 5 (2.9%) 6 (3.5%) 0 (0.0%) 1 (0.6%) 4 (2.3%) 2 (1.2%) 6 (3.5%) 112 (65.1%)	62 (17.6%) 16 (4.5%) 14 (4.0%) 1 (0.3%) 0 (0.0%) 9 (2.5%) 14 (4.0%) 15 (4.2%) 222 (62.9%)	98 (18.7%) 21 (4.0%) 20 (3.8%) 1 (0.2%) 1 (0.2%) 13 (2.5%) 16 (3.0%) 21 (4.0%) 334 (63.6%)	0.501
**Hispanic** Yes No Decline to answer Missing	11 (6.4%) 49 (28.5%) 1 (0.6%) 111 (64.5%)	42 (11.9%) 87 (24.6%) 3 (0.8%) 221 (62.6%)	53 (10.1%) 136 (25.9%) 4 (0.8%) 332 (63.2%)	0.230
**US-born** Yes No Missing	43 (25.0%) 18 (10.0%) 111 (65.0%)	87 (24.6%) 44 (12.5%) 222 (62.9%)	130 (24.8%) 62 (11.8%) 333 (63.4%)	0.800
**Education** Less than high school Some high school Completed high school Job training after high school Some college, no degree Associate degree College degree Some graduate school Graduate degree Missing	2 (1.2%) 1 (0.6%) 1 (0.6%) 1 (0.6%) 7 (4.1%) 4 (2.3%) 22 (12.8%) 1 (0.6%) 22 (12.8%) 111 (64.5%)	3 (0.8%) 4 (1.1%) 16 (4.5%) 7 (2.0%) 14 (4.0%) 8 (2.3%) 37 (10.5%) 2 (0.6%) 40 (11.3%) 222 (62.9%)	5 (1.0%) 5 (1.0%) 17 (3.2%) 8 (1.5%) 21 (4.0%) 12 (2.3%) 59 (11.2%) 3 (0.6%) 62 (11.8%) 333 (63.4%)	0.498
**Health insurance** Private Public None Missing	136 (79.1%) 36 (20.9%) 0 (0%) 0 (0%)	219 (62.0%) 125 (35.4%) 2 (0.6%) 7 (2.0%)	355 (67.6%) 161 (30.7%) 2 (0.4%) 7 (1.3%)	< 0.001
**First birth** Yes No Missing	95 (55.2%) 71 (41.3%) 6 (3.5%)	202 (57.2%) 138 (39.1%) 13 (3.7%)	297 (56.6%) 209 (39.8%) 19 (3.6%)	0.891
**Mode of delivery** Vaginal birth Cesarean birth	128 (74.4%) 44 (25.6%)	238 (67.4%) 115 (32.6%)	366 (69.7%) 159 (30.3%)	0.128
**Survey language** English Spanish	168 (97.7%) 4 (2.3%)	342 (96.9%) 11 (3.1%)	510 (97.1%) 15 (2.9%)	0.842

A total of 235 comments were analyzed and all were mapped to the 18 predefined domains (Table 2). The inter-rater reliability between the 2 coders was assessed using Cohen’s Kappa, which was calculated to be 0.92, indicating a high level of agreement. Such complete mapping supported the content validity of CBEX because all issues raised by respondents were included in the pre-defined CBEX domains. However, qualitative responses highlighted previously unexplored topics in the following 4 domains: labor management, pain management, empathy and respect, and newborn feeding.

**Table 2:** Number of respondent comments mapped to existing CBEX Domains^1^*N* = 235

CBEX Domains	CBEX Items	*N* (%)
Clinical	Provider competence; safety; preterm labor; intrapartum complications; indication for cesarean delivery; maternal and newborn clinical outcomes; additional maternal/neonatal hospitalization	9 (3.8%)
Communication	Communication with providers regarding labor and delivery, and regarding newborn	20 (8.5%)
Confidence	Confidence/self-efficacy	0 (0.0%)
Continuity of Care	Continuity of care/care coordination; provider availability	10 (4.3%)
Decision Making	Decision making and birth plans; maternal control	11 (4.7%)
Empathy and Respect	Cultural competence; discrimination; provider empathy; provider support; respect/privacy	45 (19.1%)
Intervention in labor	Labor interventions; food and drink in labor	3 (1.3%)
Labor Management	Hospital admission; labor management; labor and birth positions	14 (6.0%)
Location^2^	Childbirth location	0 (0.0%)
Mental Health	Anxiety/fear/worry; depression; maternal psychological issues	0 (0.0%)
Newborn	Newborn/ newborn care; neonatal intensive care unit (NICU); nursery environment	6 (2.6%)
Newborn Feeding	Breastfeeding/bottle feeding	14 (6.0%)
Pain Assessment	Labor pain assessment; labor pain expectations	2 (0.9%)
Pain Management	Cesarean delivery anesthesia; epidural; labor pain management	20 (8.5%)
Postpartum	Postpartum care; postpartum environment; postpartum long-term issues; postpartum work intention	16 (6.8%)
Route of Delivery	Route of delivery; vacuum/forceps; vaginal birth after cesarean (VBAC); cesarean delivery anxiety	3 (1.3%)
Summary Measures	Cesarean delivery experience; negative experience; overall experience	49 (20.9%)
Support	Labor social support; labor teaching; nursing support; partner support	13 (5.5%)
Total		235 (100.0%)

1. The original CBEX survey (antepartum and postpartum) was developed by the Childbirth Patient-Reported Outcomes Partnership and included items from 19 childbirth domains; in shortening the survey, items from the Parenting domain were excluded. The remaining 18 domains are represented here

2. All respondents gave birth at a hospital

The labor management domain captured information about the patient’s desire to choose birthing position and types of labor props (e.g., labor tub, birth ball, birth stool) and to avoid interventions (e.g., pitocin, intravenous catheter [IV], episiotomy, and operative vaginal delivery). We learned that not only were patients concerned about avoiding an induction of labor but that they expressed that they felt pressured to be induced. This concept is similar to that of patients having feeling pressured to have a cesarean delivery. For example, patients made the following comments.

I wish that patients wouldn’t be pressured into inducing as early as 37 weeks.

I felt pressured into an induction and had a failed epidural and vaginal birth which led to an emergency c section.

The pain management domain captured information about options for managing pain in labor, e.g., walking, massage, and epidural use. Patient responses highlighted specific issues related to epidurals, such as epidural effectiveness, timing, dosage, and patient education about what to expect with an epidural. The following patient quotes illustrate this range of variation.

The Epidural was too strong and was my major source of anxiety.

I was not told I was being administered Fentanyl as part of the epidural, and honestly that was the worst part, it made me feel as though if I closed my eyes I was going to die. I had no pain after the epidural but felt horrible and would have preferred to have been given the option.

The empathy and respect domain captured information about respecting patient spiritual and cultural beliefs, receiving reassurance and comfort, and providing adequate space and food for the support person. We learned that there were specific issues related to student and resident interactions with patients that contributed to feelings of discomfort and disrespect. The following quotes highlight these exchanges.

Unfortunately, with a couple residents they seemed to lead more with ego than humanity, forgetting that the patient is a person first, a learning opportunity second.

The doctor he brought in to assist him was dismissive of me, said he’d rather be outside than in the delivery room with me, and was disrespectful (i.e., he told me to smile during labor).

The newborn feeding domain captured information regarding newborn feeding plans, practical support about newborn feeding, and the level of breastfeeding encouragement the patient received from the care team. We learned that patients felt “rough handled” by nurses during lactation consults, for example, some proceeding without getting permission from the patient or being forceful in their teaching techniques. The following patient quotes highlight these types of encounters.

There was a nurse who grabbed my nipple to check for milk without my permission. I felt a little violated. She was very forceful.

The nurse brought the baby and without even asking grabbed my breast and started trying to make the baby latch on.

## Discussion

### Principal Findings

CBEX was designed to capture antepartum values and preferences (Part 1) and postpartum patient-reported experiences (Part 2) across 18 domains, including communication, decision-making, empathy and respect, pain management and newborn care. First, this study evaluated the content validity of these domains. We were able to map all the postpartum patient comments to existing CBEX domains, confirming its representativeness. This is important because it ensures that the tool comprehensively captures the key areas of patient experience relevant to quality of care and patient satisfaction. Second, we analyzed the narrative responses to assess for any new topics, and identified 4 new concerns within the domains of labor management, pain management, empathy and respect, and newborn feeding. Third, these 4 topics were further explored with our Partnership, and together we developed additional CBEX items to fill this data gap. For example, within the domain of empathy and respect, patients will be asked a series of items specific to student or resident involvement. The expansion of CBEX domains to include these new items will contribute to a more comprehensive hospital report of patient experience data used to prioritize quality improvement initiatives for improving hospital practice.

### Clinical Implications

The 4 new topics identified have important clinical implications. Perceived pressure from a clinician to induce labor or deliver by cesarean affects one-fifth of birthing people (Jou et al., 2015). Birthing people express concerns about the lack of information regarding the induction procedure, frustration with scheduling inductions, and feeling like they are not in control (Declercq et al., 2020). Since the ARRIVE trial (A Randomized Trial of Induction Versus Expectant Management), a significant proportion of induced labors are elective (Nethery et al., 2023). Importantly, the feeling of being pressured into an induction has an impact on patients’ perceptions of the labor and birthing experience (Nethery et al., 2023).

Regarding pain management, birthing people experience variable and unpredictable degrees of pain and pain tolerance during labor and birth. This may be due to a combination of factors including those that may be physical (e.g., fetal presentation), psychological (e.g., fear or anxiety), or societal (e.g., cultural beliefs) (Longinus, Omiepirisa Yvonne, & Subhamay, 2012). Approximately 73% of birthing people use neuraxial analgesia for labor and delivery in the US (Butwick et al., 2018). However, epidural analgesia for pain management neither guarantees adequate pain control nor does it ensure an optimal birth experience (Hidaka & Callister, 2012; Smith et al., 2021). This may be due to factors such as previous failed epidural, cervical dilatation > 7 cm and insertion by a trainee (Agaram et al., 2009). Complicating this is that the decision to use an epidural often occurs when patients are in severe pain, resulting in heightened vulnerability, anxiety, pain, and fear, which can impede a comprehensive understanding of the risks and side effects (e.g., decrease in blood pressure, headache) and benefits of epidural use (e.g., pain relief) (Borrelli et al., 2020). Our data support the need to provide patients with adequate prenatal education about the risks and benefits of epidurals for pain management (Wada et al., 2019), which may include the use of a decision aid to manage expectations about pain relief (Shishido et al., 2020).

Birthing people experience variability in the degree of empathy and respect expressed by students and residents, resulting in reluctance and hesitancy to include them as members of their care team. Patients’ reluctance to accept student and resident participation is influenced by concerns regarding adequate student supervision, training level, desire for seasoned medical personnel, previous negative experiences, and gender (Subki et al., 2018; Woolner & Cruickshank, 2015). A 2001 study estimated that while 62% of antenatal patients are willing to receive care from medical students, only 54% can correctly identify them (Grasby & Quinlivan, 2001). Our data suggests that student and resident involvement can have an impact on the childbirth experience. Additional effort must be made to inform, educate, and clarify the role of the medical student and resident to the patient, and to inform, educate, and emphasize the importance of demonstrating respect and empathy by the trainee towards the patient as medical education is a vital component of the healthcare system.

Within the domain of newborn feeding, it is well documented that using lactation consultants and counselors increases the number of birthing people who initiate breastfeeding (Massare et al., 2023; Patel & Patel, 2016). However, breastfeeding patients have identified many areas for care improvement, including continuity of care and unsupportive interactions such as intrusive behavior, inadequate assessments, and limited or contradictory guidance (Hong et al., 2003). These behaviors are especially important because breastfeeding people tend to rate social support as more important than health services support as incentive to continue breastfeeding (McInnes & Chambers, 2008). Additionally, when patients encounter breastfeeding challenges, it is essential that support be accessible without being imposing and that it be provided with sensitivity, prioritizing the emotional well-being of both the infant and mother (Chaput et al., 2015).

### Strengths and Limitations

The primary strength of this study was the analysis of all 235 respondent comments. By mapping all comments (versus a sample), we maximized our opportunity to identify any additional issues or topics. We also grounded the analysis in a previously validated list of domains that was familiar to both reviewers. We recognize the inherent limitations in qualitative research, including potential for researcher subjectivity, variation in researcher analysis, and lack of replicability (Anderson, 2010). Understanding these limitations, a-priori, we utilized a deductive coding technique, which supported consistent mapping of comments to domains. Another inherent limitation of this research design is non-response bias. Another limitation is the high number of missing demographic information in Table 1. Participants were encouraged, but not required, to provide responses to these self-reported survey data, resulting in approximately 64% missing data consistently across race, ethnicity, US born, education, and insurance.

## Conclusions

This paper describes findings from a qualitative analysis of postpartum CBEX survey data, confirms its content validity, and provides additional details to improve hospital practice. CBEX survey data is currently being utilized in hospitals to inform childbirth-specific quality improvement initiatives. By capturing detailed voluntary participant responses, CBEX provides the opportunity to document and explore nuanced aspects of the childbirth experience and subtleties that may be contributing to maternal dissatisfaction (Fink et al., 2023; Mohamoud et al., 2023).

## Data Availability

Complete survey is published and available on the Patient-Centered Outcome Research Institute Webpage: https://www.pcori.org/research-results/2014/developing-item-bank-survey-questions-measure-womens-experiences-childbirth-hospitals. Qualitative data will be made available upon reasonable request.

## Code Availability

Qualitative coding presented in Table 1.
